# Differences in Online Consumer Ratings of Health Care Providers Across Medical, Surgical, and Allied Health Specialties: Observational Study of 212,933 Providers

**DOI:** 10.2196/jmir.9160

**Published:** 2018-05-09

**Authors:** Timothy Daskivich, Michael Luu, Benjamin Noah, Garth Fuller, Jennifer Anger, Brennan Spiegel

**Affiliations:** ^1^ Division of Urology Cedars-Sinai Medical Center Los Angeles, CA United States; ^2^ Cedars-Sinai Center for Outcomes Research and Education Cedars-Sinai Medical Center Los Angeles, CA United States; ^3^ Samuel Oschin Comprehensive Cancer Institute Cedars-Sinai Medical Center Los Angeles, CA United States; ^4^ Department of Medicine Division of Health Services Research Cedars-Sinai Health System Los Angeles, CA United States; ^5^ Department of Health Policy and Management UCLA Fielding School of Public Health Los Angeles, CA United States

**Keywords:** online ratings, consumer ratings, patient satisfaction, digital health, telemedicine

## Abstract

**Background:**

Health care consumers are increasingly using online ratings to select providers, but differences in the distribution of scores across specialties and skew of the data have the potential to mislead consumers about the interpretation of ratings.

**Objective:**

The objective of our study was to determine whether distributions of consumer ratings differ across specialties and to provide specialty-specific data to assist consumers and clinicians in interpreting ratings.

**Methods:**

We sampled 212,933 health care providers rated on the Healthgrades consumer ratings website, representing 29 medical specialties (n=128,678), 15 surgical specialties (n=72,531), and 6 allied health (nonmedical, nonnursing) professions (n=11,724) in the United States. We created boxplots depicting distributions and tested the normality of overall patient satisfaction scores. We then determined the specialty-specific percentile rank for scores across groupings of specialties and individual specialties.

**Results:**

Allied health providers had higher median overall satisfaction scores (4.5, interquartile range [IQR] 4.0-5.0) than physicians in medical specialties (4.0, IQR 3.3-4.5) and surgical specialties (4.2, IQR 3.6-4.6, *P*<.001). Overall satisfaction scores were highly left skewed (normal between –0.5 and 0.5) for all specialties, but skewness was greatest among allied health providers (–1.23, 95% CI –1.280 to –1.181), followed by surgical (–0.77, 95% CI –0.787 to –0.755) and medical specialties (–0.64, 95% CI –0.648 to –0.628). As a result of the skewness, the percentages of overall satisfaction scores less than 4 were only 23% for allied health, 37% for surgical specialties, and 50% for medical specialties. Percentile ranks for overall satisfaction scores varied across specialties; percentile ranks for scores of 2 (0.7%, 2.9%, 0.8%), 3 (5.8%, 16.6%, 8.1%), 4 (23.0%, 50.3%, 37.3%), and 5 (63.9%, 89.5%, 86.8%) differed for allied health, medical specialties, and surgical specialties, respectively.

**Conclusions:**

Online consumer ratings of health care providers are highly left skewed, fall within narrow ranges, and differ by specialty, which precludes meaningful interpretation by health care consumers. Specialty-specific percentile ranks may help consumers to more meaningfully assess online physician ratings.

## Introduction

Health care consumers are increasingly using commercial online consumer ratings websites to rate and select medical providers. A recent study of 600 randomly selected physicians from 3 metropolitan areas in the United States revealed that 66% of physicians had at least one rating across several popular online ratings websites, with a median of 7 reviews per physician [1]. Patients also appear to strongly trust these data. Even as early as 2012, a survey found that 59% of US adults believed that online ratings websites were “somewhat important” or “very important” in selecting a physician [2]. And perhaps more strikingly, a survey of 1000 surgical outpatients from the Mayo Clinic in Rochester, MN, USA, found that 75% of patients would choose a physician and 88% would avoid seeing a physician based on ratings data alone [3]. Payers and health systems are also now including consumer ratings in their online tools for patients, which provides tacit endorsement for the ratings’ validity in comparing doctors [4,5]. The extent of consumers’ use of online ratings suggests that these data have important implications for the use of health services and may even have downstream effects on health.

Yet, despite the public’s strong interest and trust in online physician ratings, interpretation of numeric physician ratings is difficult due to the lack of established benchmarks for scoring and the normalization of results for meaningful interpretation [6]. The most popular online consumer ratings websites use a 5-star Likert-type scale to rate providers, often reported as an overall score and sometimes across domains of performance categories. While consumers may assume that higher scores (ie, scores of 4 and 5) indicate above-average performance, this may not be so if ratings are not normally distributed [7]. In fact, the percentile rank for a given star rating may differ drastically based on how scores are distributed, such that a seemingly high score may indicate average or even below-average performance [8]. Furthermore, it is possible that distributions of scores may differ by specialty due to the varying perceptions of performance associated with patients’ specific needs and the services provided by different specialties.

In this study, we sought to determine how online provider consumer ratings are distributed across medical, surgical, and allied health professions and whether score distributions differ across individual specialties in the United States. To address this question, we created a novel dataset consisting of over 2.7 million reviews of approximately 830,000 providers reviewed in both the US Centers for Medicare & Medicaid Services (CMS) Physician Compare [9] and the Healthgrades online consumer rating websites [10]. Our objectives were to (1) describe the distribution of quantitative overall satisfaction scores in aggregate and across provider specialties, (2) assess whether these distributions were normal, (3) quantify how overall satisfaction scores related to percentile rank across provider specialties, and (4) provide specialty-specific lookup tables showing percentile rank by overall satisfaction score. We hypothesized that overall satisfaction scores would be strongly left skewed toward higher scores across all specialties, such that seemingly high scores would be associated with a relatively low percentile rank. Lookup tables translating overall satisfaction scores into specialty-specific percentile ranks would allow for consumer ratings data to be communicated to patients in a more meaningful and accurate manner.

## Methods

### Data Source and Participants

We sampled online consumer reviews for providers in the United States from the Healthgrades website. Our dataset consisted of all reviews up to March 31, 2017, of 830,308 health care providers. We aggregated data at the provider level to calculate an average rating for each provider across a variety of metrics: overall satisfaction, level of trust in provider’s decisions, how well the provider explains medical conditions, how well the provider listens and answers questions, and spending the appropriate amount of time with patients. We collected data on the following office metrics: ease of scheduling urgent appointments, the office environment, staff friendliness and courteousness, and total wait time. We also captured data on the number of reviews per provider. We linked these data to demographic information publicly available on the CMS Physician Compare website [9] using national provider identification numbers to capture medical specialty, region, sex, and year of graduation from medical school. Allied health specialties were defined as health professions distinct from medicine and nursing. We excluded providers with no data on overall patient satisfaction (n=345,862); no data on primary specialty (n=11,762); fewer than 4 reviews (the median number of reviews per provider in the overall dataset; n=255,202); and providers in nursing specialties (n=4549). Our final analytic sample consisted of 212,933 providers.

### Variables

#### Consumer Ratings

The Healthgrades website asks consumers to rate providers on a 5-star Likert-type scale across the domains of patient experience listed above. Individual ratings are quantized at the ordinal level, though average ratings are reported to the 10th decimal place. Average ratings for each domain were aggregated at the provider level.

#### Covariates

We collected information on US geographical region (New England, Mid-Atlantic, East North Central, West North Central, South Atlantic, East South Atlantic, West South Central, Mountain, and Pacific), sex (male, female), and graduation year (in deciles of graduation year) using linked data from the CMS Physician Compare website.

### Statistical Analysis

We first compared our sample characteristics across medical, surgical, and allied health specialties using chi-square analysis for categorical variables and the Wilcoxon-Mann-Whitney test for continuous variables.

To assess whether consumer ratings scores followed a normal distribution, we created histograms showing the distribution of overall patient satisfaction scores across the medical, surgical, and allied health specialties, along with individual specialties. We then assessed the divergence from normality by determining skewness and kurtosis. Skewness is a measure of symmetry of the distribution of scores, with a negative skew indicating a preponderance of higher scores and a positive skew indicating a preponderance of lower scores; normal distributions generally have skewness values between –0.5 and 0.5. Kurtosis is a measure of the tailedness of the distribution compared with the standard normal distribution; positive kurtosis values indicate a heavier tail and a higher propensity for outliers, while negative values indicate a lighter tail. Normal distributions generally have kurtosis values around 0. We performed bootstrap resampling with 100 replicates to obtain bootstrap confidence intervals for skewness and kurtosis across groupings of specialties using the basic bootstrap method.

We then calculated the percentile rank for overall patient satisfaction scores within individual specialties and visualized them in a scatterplot figure. We used a locally weighted scatterplot smoother to visualize percentile rank by overall patient satisfaction scores across groupings of specialties.

We used *P*<.05 to denote the statistical significance of 2-sided tests. All statistical analyses were performed in R version 3.4.0 (R Foundation for Statistical Computing). The Cedars-Sinai Institutional Review Board approved this study.

## Results

Our analytic sample comprised 212,933 providers across 29 medical specialties (n=128,678), 15 surgical specialties (n=72,531), and 6 allied health professions (n=11,724; Table 1). Most providers in our sample were male (156,556/212,933, 73.52%), were from the South region (80,751/212.933, 37.92%), and graduated from medical school after 1985 (146,246/212,933, 68.68%). More of the providers in medical specialties than in surgical specialties or allied health providers were women (*P*<.001). Allied health providers graduated later than those in the medical or surgical specialties (*P*<.001).

**Figure 1:**
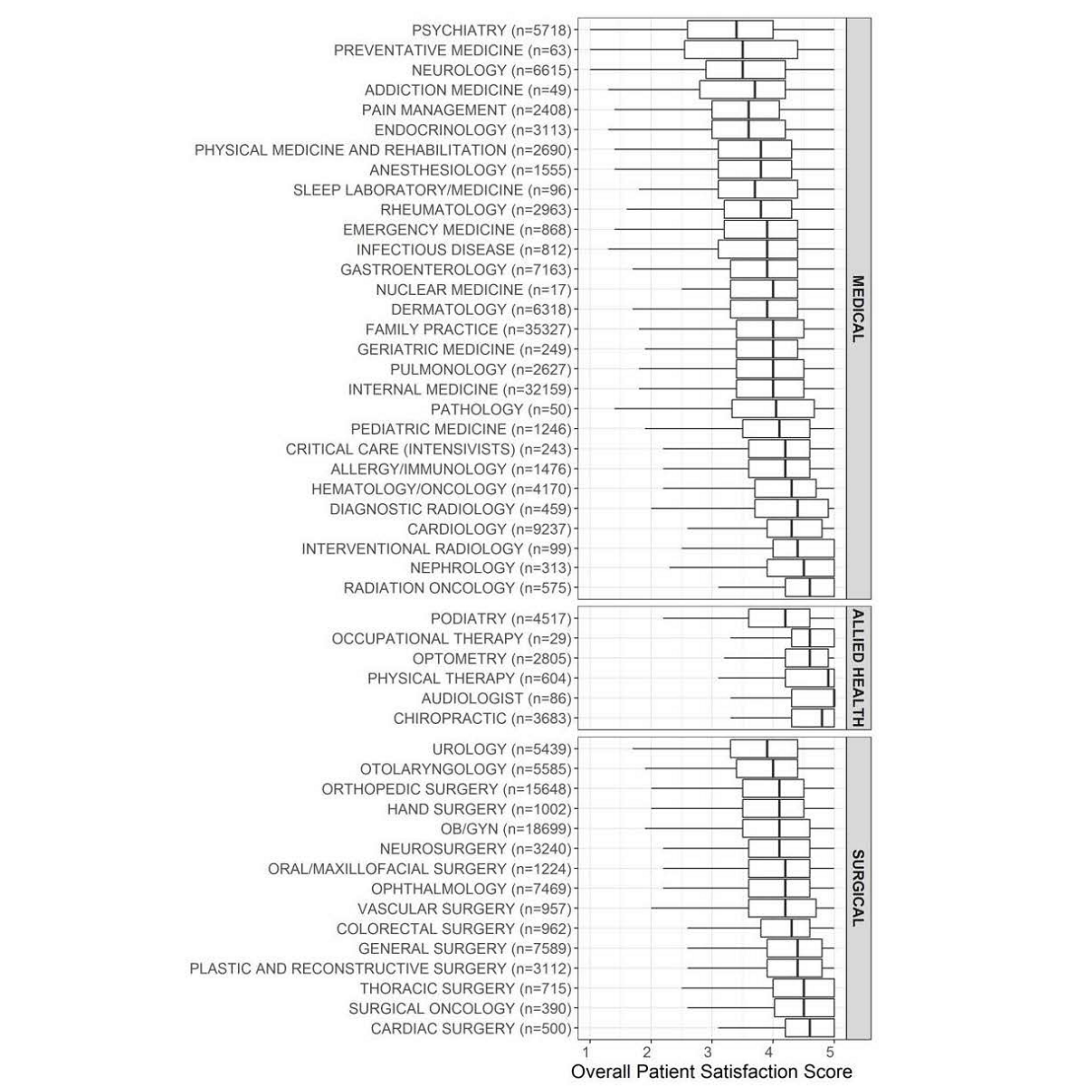
Boxplots depicting the distribution of mean overall satisfaction ratings by provider specialty. OB/GYN: obstetrics and gynecology.

Median overall satisfaction scores differed significantly by provider specialty (Figure 1). Allied health providers had higher median overall satisfaction scores (4.5, interquartile range [IQR] 4.0-5.0) than physicians in medical (4.0, IQR 3.3-4.5) and surgical specialties (4.2, IQR 3.6-4.6; *P*<.001). There were also significant differences in median scores across subdomains of physician metrics and office and staff performance metrics by specialty (*P*<.001; Table 1).

**Figure 2:**
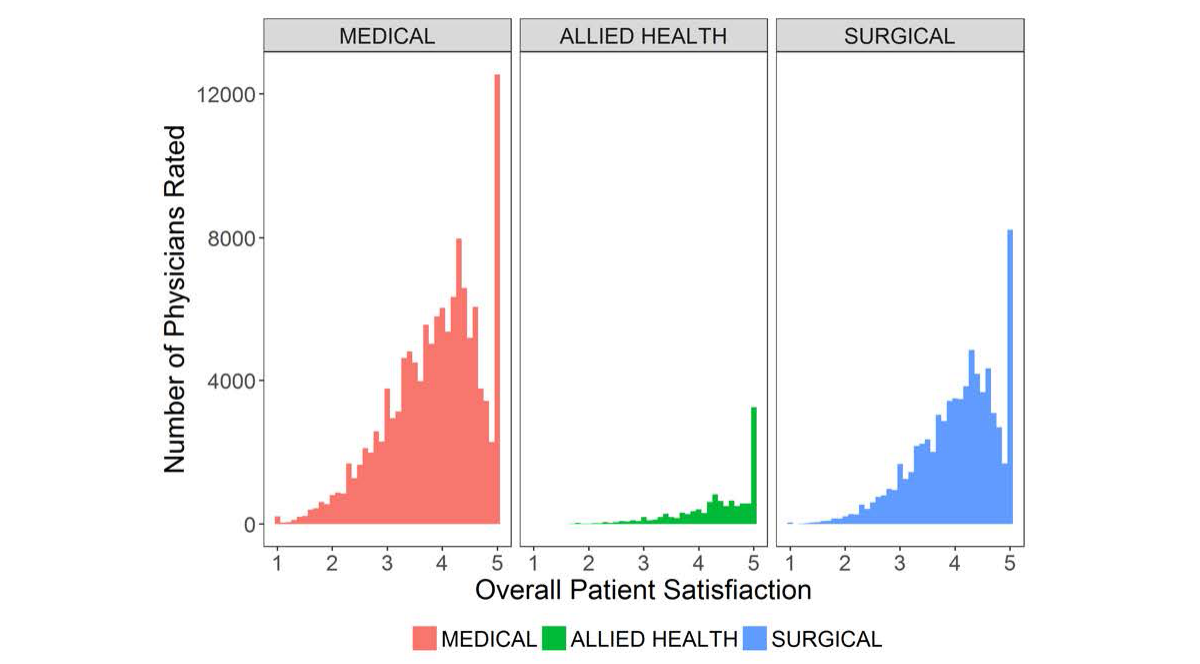
Frequency of mean overall patient satisfaction scores across medical, surgical, and allied health providers.

Measures of normality also differed by provider specialty. Overall satisfaction scores were highly left skewed for all provider groups, but skewness differed by specialty (Figure 2). Allied health providers had the largest negative skewness (ie, preponderance of higher scores; –1.23, 95% CI –1.280 to –1.181), compared with physicians in the surgical specialties (–0.77, 95% CI –0.787 to –0.755) and medical specialties (–0.64, 95% CI –0.648 to –0.628). Distributions of overall satisfaction scores had variable kurtosis across specialties; allied health providers had the largest positive kurtosis (ie, heavy-tailed with more outliers; 1.30, 95% CI 1.109-1.531), compared with physicians in the surgical specialties (0.26, 95% CI 0.206-0.315) and medical specialties (–0.07, 95% CI –0.101 to –0.041).

**Figure 3:**
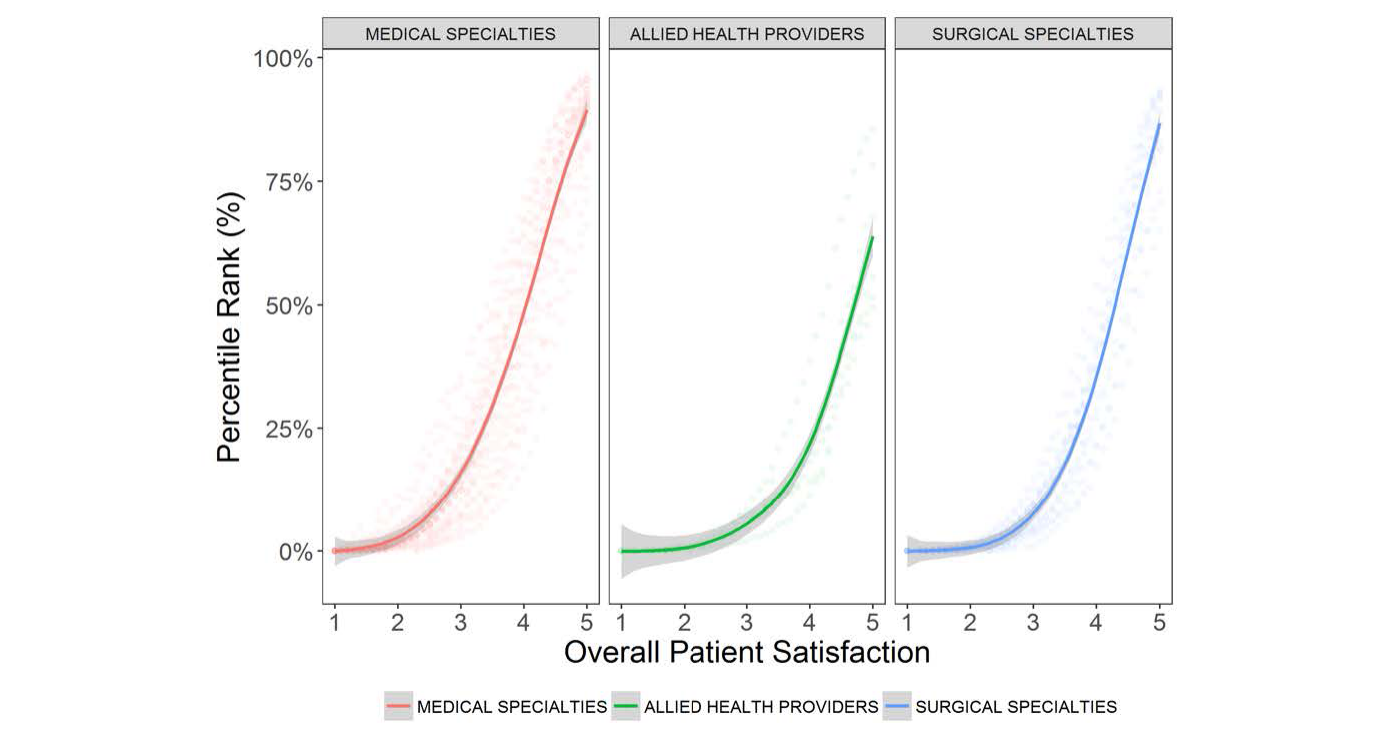
Percentile rank versus mean overall patient satisfaction for allied health, medical specialties, and surgical specialties. Percentile rank associated with overall patient satisfaction was first calculated within individual specialties (eg, internal medicine, podiatry, urology) as represented by scatter dots. Lines represent the locally weighted scatterplot smoothing line smoother best fit for percentile rank among specialty groupings (ie, medical, surgical, allied health). Gray bars around lines represent 95% confidence intervals for percentile rank estimates among specialty groupings.

**Figure 4:**
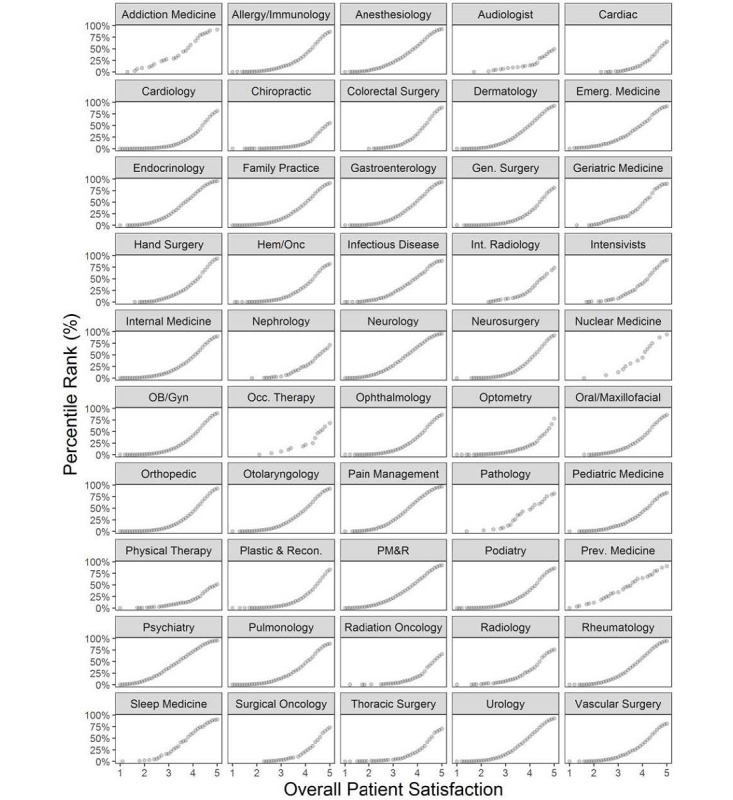
Percentile rank versus mean overall patient satisfaction across individual specialties. Emerg: emergency; Gen: general; Hem/Onc: hematology and oncology; Int: interventional; OB/GYN: obstetrics and gynecology; Occ: occupational; PM&R: physical medicine and rehabilitation; Prev: preventative; Recon: reconstructive.

To communicate consumer ratings data in a way that accounts for differences in distribution of overall satisfaction scores across specialties, we calculated the percentile rank for overall satisfaction scores by provider specialty. This information allows for translation of a provider’s overall satisfaction rating into a percentile ranking compared with others in their specialty. Consistent with the left skew of the data, percentile ranks were low for seemingly high overall satisfaction scores across all specialties. Percentile rank for overall satisfaction varied across allied health, medical specialties, and surgical specialties for scores of 2 (0.7%, 2.9%, 0.8%, respectively), 3 (5.8%, 16.6%, 8.1%), 4 (23.0%, 50.3%, 37.3%), and 5 (63.9%, 89.5%, 86.8%; Figure 3). As a point of reference, if overall satisfaction scores were normally distributed, the 50th percentile would occur at a score of 3. Percentile rank for overall satisfaction scores also differed substantially by individual specialties, reflecting their variable deviation from normality (Figure 4). A Web-based tool for translating overall satisfaction ratings to specialty-specific percentile rankings is available [11].

**Table 1:** Sample characteristics.

Characteristics		Overall (n=212,933)	Medical specialties (n=128,678)	Allied health providers (n=11,724)	Surgical specialties (n=72,531)	*P* value^a^
**Region, n (%)**						.02
	Northeast	45,616 (21.42)	27,385 (21.28)	2654 (22.64)	15,577 (21.48)	
	Midwest	44,069 (20.70)	26,629 (20.69)	2389 (20.38)	15,051 (20.75)	
	South	80,751 (37.92)	48,779 (37.91)	4380 (37.36)	27,592 (38.04)	
	West	41,197 (19.35)	25,121 (19.52)	2234 (19.05)	13,842 (19.08)	
	Not available	1300 (0.61)	764 (0.59)	67 (0.57)	469 (0.65)	
**Division, n (%)**						<.001
	New England	12,345 (5.80)	7517 (5.84)	615 (5.25)	4213 (5.81)	
	Mid-Atlantic	33,271 (15.63)	19,868 (15.40)	2039 (17.39)	11,364 (15.67)	
	East North Central	32,797 (15.40)	20,058 (15.59)	1728 (14.74)	11,011 (15.18)	
	West North Central	11,272 ( 5.29)	6571 (5.11)	661 (5.64)	4040 (5.57)	
	South Atlantic	45,537 (21.39)	27,829 (21.63)	2570 (21.92)	15,138 (20.87)	
	East South Atlantic	11,840 (5.56)	7008 (5.45)	575 (4.90)	4257 (5.87)	
	West South Central	23,374 (10.98)	13,942 (10.83)	1235 (10.53)	8197 (11.30)	
	Mountain	15,012 (7.05)	8816 (6.85)	1043 (8.90)	5153 (7.10)	
	Pacific	26,185 (12.30)	16,305 (12.67)	1191 (10.16)	8689 (11.98)	
	Not available	1300 (0.61)	764 (0.59)	67 (0.57)	469 (0.65)	
**Sex, n (%)**						<.001
	Female	56,377 (26.48)	38,293 (29.76)	2592 (22.11)	15,492 (21.36)	
	Male	156,556 (73.52)	90,385 (70.24)	9132 (77.89)	57,039 (78.64)	
**Graduation year, n (%)**						<.001
	1945-1954	57 (0.02)	47 (0.04)	2 (0.02)	8 (0.01)	
	1955-1964	1579 (0.74)	1025 (0.80)	18 (0.15)	536 (0.74)	
	1965-1974	13,475 (6.33)	8360 (6.50)	314 (2.68)	4801 (6.62)	
	1975-1984	47,738 (22.42)	29,744 (23.12)	2124 (18.12)	15,870 (21.88)	
	1985-1994	64,498 (30.29)	39,094 (30.38)	3128 (26.68)	22,276 (30.71)	
	1995-2004	61,338 (28.81)	36,279 (28.19)	3855 (32.88)	21,204 (29.23)	
	2005-2014	20,349 (9.56)	11,665 (9.07)	2215 (18.89)	6469 (8.92)	
	2015-2016	61 (0.03)	11 (0.01)	40 (0.34)	10 (0.01)	
	Not available	3838 (1.80)	2453 (1.91)	28 (0.24)	1357 (1.87)	
**Physician performance metrics, median (IQR^b^)**						
	Overall patient satisfaction	4.10 (3.40-4.60)	4.00 (3.30-4.50)	4.50 (4.00-5.00)	4.20 (3.60-4.60)	<.001
	Level of trust in provider’s decisions	4.20 (3.60-4.60)	4.10 (3.50-4.60)	4.60 (4.10-5.00)	4.30 (3.70-4.70)	<.001
	How well provider explains medical condition(s)	4.20 (3.60-4.60)	4.10 (3.50-4.60)	4.60 (4.10-5.00)	4.30 (3.70-4.70)	<.001
	How well provider listens and answers questions	4.20 (3.60-4.60)	4.10 (3.50-4.60)	4.60 (4.10-5.00)	4.20 (3.70-4.60)	<.001
	Spends appropriate amount of time with patients	4.20 (3.60-4.60)	4.10 (3.50-4.60)	4.60 (4.10-5.00)	4.20 (3.70-4.60)	<.001
**Office and staff performance metrics, median (IQR)**						
	Ease of scheduling urgent appointments	4.20 (3.60-4.60)	4.00 (3.50-4.50)	4.60 (4.20-4.90)	4.30 (3.80-4.60)	<.001
	Office environment, cleanliness, comfort	4.30 (3.90-4.70)	4.30 (3.80-4.60)	4.60 (4.20-4.90)	4.40 (4.00-4.70)	<.001
	Staff friendliness and courteousness	4.20 (3.70-4.60)	4.10 (3.60-4.50)	4.60 (4.20-4.90)	4.30 (3.90-4.70)	<.001
**Total wait time (waiting and exam rooms; minutes), n (%)**						<.001
	<10	31,177 (14.64)	17,412 (13.53)	5826 (49.69)	7939 (10.95)	
	10-15	113,517 (53.31)	69,132 (53.72)	4676 (39.88)	39,709 (54.75)	
	16-30	54,412 (25.55)	32,548 (25.29)	1057 (9.02)	20,807 (28.69)	
	31-45	12,907 (6.06)	8836 (6.87)	156 (1.33)	3915 (5.40)	
	>45	908 (0.43)	740 (0.58)	8 (0.07)	160 (0.22)	

^a^*P* value calculated by Pearson chi-square test.

^b^IQR: interquartile range.

## Discussion

### Principal Findings

Online consumer ratings of health care providers are playing an increasing role in how consumers perceive and select providers. However, since online ratings lack standardized benchmarks for assessment and because ratings are not normalized, it is unclear how consumers should interpret scores. Our study showed that overall satisfaction scores are consistently left skewed, fall within narrow ranges, and have different distributions across specialties; as a result, scores that appear high might actually be in the lowest quartile of scores, effectively misleading patients about perceived quality or experience of care. Allied health specialties tend to be the least normally distributed (6/6, 100% of specialties, either moderately or highly skewed—ie, skewness greater than –0.5), followed by surgical specialties (14/15, 93% of specialties), and medical specialties (16/29, 55% of specialties). Overall satisfaction scores also fall within narrow ranges; the average IQR spanned only 1.2 stars for medical specialties and 1.0 for allied health and surgical specialties. Median overall satisfaction scores also varied across specialties, with median values ranging from 3.4 to 4.6 for medical specialties, 3.9 to 4.6 for surgical specialties, and 4.2 to 4.9 for allied health professions.

Deviations from normality and differences in score distributions (ie, median, IQR) across specialties have a substantial impact on how scores should be interpreted by consumers. First, since scores across all specialties were drastically left skewed, consumers should be aware that most scores are high, which falsely implies that most doctors are above average. We found that median values for overall satisfaction scores were 4.0, 4.2 and 4.5, and the 25th percentiles for overall satisfaction scores were 3.4, 3.5, and 4.0 for medical, surgical, and allied health professions, respectively. Given this information, a score of 3—which would be considered average if scores were normally distributed—would be exceedingly low in terms of percentile rank across all medical professions. Second, due to the narrow ranges of scores within professions, consumers should be aware that small differences in scores may represent large differences in percentile rank; for example, a difference of 0.5 stars among a surgical provider may indicate a quartile difference in percentile rank. Third, given the significant differences in median overall satisfaction score distributions across specialties, there may be even more granular differences in how scores should be interpreted for individual specialties. For example, a urologist with 4.6 stars would be at the 80th percentile among his or her peers, whereas a cardiothoracic surgeon with the same star rating would be only at the 50th percentile.

In response to these findings, there are several feasible measures that could improve the interpretability of online physician consumer ratings data. First, data should be reported in a way that accounts for its consistent left skewness and nonnormality. One option would be to report the *median* star rating for each physician as a specialty-specific percentile rank, which would reflect the nonparametric nature of the data, would reduce the impact of outliers, and would be easily interpretable [8]. Another option would be to report the frequency of ratings falling within specialty-specific quartiles of performance, which would accomplish similar goals. Second, data should be reported in a way that accounts for varying distributions across specialties and subspecialties, since our data showed that patients have different benchmarks for scoring for different health care services and types of providers. We believe that our rubric for calculating percentile rank by average overall satisfaction score for individual specialties (available in a user-friendly, Web-based format [11]) may be a useful tool for describing these data to patients in a meaningful way.

While consumer ratings data may seem trivial to health care providers who are often focused on hard end points related to health [12], it is important to note that health care consumers strongly trust these data and choose providers based on them [2,3]. Although studies have shown that numeric online consumer ratings are not related to quality or value of care [13,14], this has not dampened the public’s enthusiasm about their use. In fact, numerous surveys have shown that patients use online consumer ratings as the *sole* determinant of whether or not to see a physician in consultation over three-quarters of the time [3,15]. This underscores the need for physicians to be focused not only on technical execution of their practice but also on providing excellent customer service. If patients believe that customer service (vis-à-vis consumer ratings) is important, we as health care providers should respond by measuring it accurately, describing it meaningfully, and making it a priority in the way we practice, not by ignoring it in favor of what we feel to be more important [12,16,17]. Ultimately, measurements of quality of care and consumer ratings should be provided in tandem to help consumers understand these separate components of the patient experience [5].

### Study Limitations

Our study has some limitations. First, it is unclear whether results from the Healthgrades website are generalizable to other consumer ratings platforms, since distributions of scores may differ from platform to platform. Second, our findings may underestimate the degree of nonnormality of physician ratings due to our exclusion of providers with few ratings, since the vast majority of physicians with 1 rating had scores of 5. We decided to exclude physicians with fewer than 4 reviews (the median number of reviews in our overall sample) to ensure that average scores were representative of multiple ratings; sensitivity analyses showed little difference between distributions when we increased the threshold for the number of reviews beyond 4. Third, we cannot account for self-rating of physicians or other practices that may be used to artificially inflate consumer ratings scores; our reported scores represent distributions that would be observed in the real-life setting. Fourth, because we did not weight individual physician ratings scores by number of reviews, our reported results describe the distribution of average scores at the physician level.

### Conclusions

Online consumer ratings of physicians are an increasingly important factor in how patients perceive and select physicians. We found that scores were highly left skewed, fell within narrow ranges, and differed by specialty; this may mislead consumers into overestimating providers with seemingly high scores who are actually mediocre or poor when compared with peers in their specialty. We herein provide a Web-based tool for translating an overall satisfaction star rating into a percentile rank comparing the provider across others in his or her specialty, an approach that accounts for the skewness and specialty-specific differences in satisfaction scores. As online consumer ratings grow in popularity, consumers will no doubt demand more detailed forms of information regarding provider service, including comparisons within specialties such as we present here. We hope our work stimulates more research on how to convey consumer ratings data in a clear, fair way, given the degree to which this information affects health care consumers’ decisions.

## Abbreviations

CMS: Centers for Medicare & Medicaid Services
OB/GYN: obstetrics and gynecology
IQR: interquartile range
